# Pharmacokinetics of artemether and dihydroartemisinin in healthy Pakistani male volunteers treated with artemether-lumefantrine

**DOI:** 10.1186/1475-2875-9-275

**Published:** 2010-10-11

**Authors:** Shabana Ali, Muzammil H Najmi, Joel Tarning, Niklas Lindegardh

**Affiliations:** 1 Department of Pharmacology and Therapeutics, Army Medical College, National University of Sciences and Technology(NUST), Islamabad, Pakistan; 2 Mahidol-Oxford Tropical Medicine Research Unit, Faculty of Tropical Medicine, Mahidol University, Bangkok, Thailand; 3 Centre for Tropical Medicine, Nuffield Department of Clinical Medicine, University of Oxford, Oxford, UK

## Abstract

**Background:**

Artemether-lumefantrine is one of the most widely used anti-malarial drug combinations in the world with excellent tolerability and cure rates in adult and paediatric patients with uncomplicated falciparum malaria. The aim of this study was to evaluate the pharmacokinetics of artemether and its active metabolite, dihydroartemisinin, in healthy Pakistani volunteers.

**Methods:**

Twelve healthy male Pakistani subjects, aged 20 to 50, were recruited into the study. A fixed oral combination of artemether-lumefantrine (80-480 mg) was given as a single oral dose. Frequent blood samples were collected and artemether and dihydroartemisinin were quantified in human plasma using solid-phase extraction and liquid chromatography coupled with tandem mass spectrometry. Drug concentration-time data were evaluated with non-compartmental analysis.

**Results:**

Observed maximum concentrations (mean ± SD) of artemether and dihydroartemisinin were 184 ± 100 ng/mL and 126 ± 46 ng/mL, respectively. These concentrations were reached at 1.56 ± 0.68 hr and 1.69 ± 0.59 hr, respectively, after drug intake. The terminal elimination half-life of artemether and dihydroartemisinin were 2.00 ± 0.71 hr and 1.80 ± 0.31 hr, respectively. Apparent volume of distribution and oral clearance for artemether were estimated to 666 ± 220 L and 257 ± 140 L/hr. The same parameters were estimated to 702 ± 220 L and 269 ± 57 L/hr for dihydroartemisinin.

**Conclusions:**

The overall pharmacokinetic properties of artemether and dihydroartemisinin in healthy Pakistani subjects are comparable to healthy subjects and patients from other populations.

## Background

Malaria is one of the most important infectious diseases in the world. This parasitic disease caused almost one million deaths in 2006, mostly children under the age of 5 [1]. Artemisinin is a natural anti-malarial derived from the Chinese medicinal plant *Artemisia annua*. The artemisinin derivatives are the most effective anti-malarial drugs available today and they have been used with success in areas with multidrug resistant *Plasmodium falciparum *malaria [2-8]. These compounds give a rapid reduction in parasite biomass and consequently a rapid resolution of symptoms [9]. Artemisinin based combination therapy (ACT) is now recommended as first-line treatment worldwide and used to treat millions of malaria patients each year.

Exafal™ is a fixed-dose ACT formulation of artemether and lumefantrine, with each tablet containing 20 mg artemether and 120 mg lumefantrine. The recommended treatment is four tablets twice a day for three days (administered at 0, 8, 24, 36, 48, and 60 hr). This anti-malarial is generally well tolerated and highly efficacious in adults and children with uncomplicated falciparum malaria [2-8]. Recent reports have shown lower cure rates in pregnant women with uncomplicated falciparum malaria due to lower drug concentrations of artemether, dihydroartemisinin and lumefantrine [10-12]. Artemether is rapidly absorbed and extensively metabolized (i.e. demethylated) in vitro by the cytochrome P450 (CYP) enzymes CYP1A2, CYP2B6, CYP2C19 and CYP3A4 into dihydroartemisinin [13,14]. However, no major contribution of CYP2C19 and CYP2D6 was seen in the demethylation of artemether in healthy volunteers [14]. Dihydroartemisinin (geometric mean IC50-value of 1.2 ng/mL) is intrinsically more active than artemether (geometric mean IC50-value of 4.8 ng/mL) as an anti-malarial when investigated in fresh clinical falciparum isolates on the Thai-Burmese border [15].

Different human CYP content and isoform capacity has generally been attributed to genetic factors [16]. This contributes to the wide variety in drug metabolism capacity among different individuals and populations. This is especially true for CYP3A4 and CYP2 D and drugs cleared by these isoforms might therefore be expected to show highly variable pharmacokinetics [17]. Artemether and dihydroartemisinin show large inter-individual variability in pharmacokinetic parameters. This may in some part be explained by genetic differences in different individuals and populations. Inter-ethnic variability might therefore be an important factor when accounting for inter-individual variability in drug response [18]. The pharmacokinetics of artemether and dihydroartemisinin is described in a number of different populations, such as Caucasians [19], Thais [20], Chinese [21] and Malaysians [22]. However, the pharmacokinetics of artemether and dihydroartemisinin has not yet been studied in a Pakistani population.

The objective of this study was to characterize the pharmacokinetics of artemether and dihydroartemisinin in healthy Pakistani male subjects after a single oral administration of the standard fixed artemether-lumefantrine anti-malarial combination treatment.

## Methods

### Study site and ethical approval

Twelve healthy Pakistani male subjects aged 20 to 50 years were enrolled in this study, conducted at the Department of Pharmacology and Therapeutics, Centre for Research in Experimental and Applied Medicine (CREAM), Army Medical College, Rawalpindi, Pakistan. The study protocol was approved by the Research Ethics Committee at the National University of Sciences and Technology, Islamabad, Pakistan. The purpose of the study, potential discomfort and adverse events were carefully explained to the volunteers in their own language and written consent was obtained. Physical and laboratory examinations were performed which included screening for cardiovascular, renal, hepatic and/or gastrointestinal disorders. The exclusion criteria were clinically significant ECG abnormalities, smoking, known renal or hepatic dysfunction. None of the volunteers had a recent history (< 3 months) of taking anti-malarials or concurrent medications. Only those who wilfully consented to participate and that fulfilled all of the inclusion criteria and none of the exclusion criteria were enrolled in the study.

### Study drug

Four tablets of artemether-lumefantrine (Exafal™: each tablet contains 20/120 mg artemether/lumefantrine), produced by Novartis, Karachi, Pakistan were administered as a single oral dose. The drug was taken with a glass of milk (250 mL) after an overnight fast. The volunteers were provided a high fat traditional breakfast (i.e. Cheese omelette, fried wheat flour bread, meat gravy, and lentils) one hr after the drug administration. Relative bioavailability of artemether and lumefantrine has been demonstrated to increase with more than two-fold and five-fold, respectively, when administered with a high fat (> 1.2 g) meal compared with that in fasted individuals [23,24].

### Plasma sample collection

Blood samples for drug quantification were taken from an indwelling venous canula during the first 12 hr and thereafter by direct venipuncture. Five milliliters of blood were collected on each occasion in lithium-heparinized tubes and centrifuged without delay at 2,200 g for five minutes at 4°C. The plasma was kept in polypropylene screw capped tubes at -80°C until analysed. Blood samples were taken from each individual pre-dose (0 hr) and at 0.25, 0.5, 0.75, 1, 1.25, 1.5, 1.75, 2, 2.5, 3, 4, 6, 8, 12, and 24 hr post-dose.

### Drug analysis

This study was conducted in January 2008 and plasma samples were sent on dry-ice to the Clinical Pharmacology Laboratory at the Faculty of Tropical Medicine, Mahidol University, Bangkok, Thailand for artemether and dihydroartemisinin quantification (June 2008). Artemether and dihydroartemisinin are stable for at least two years at -80°C (in-house validation data and also to be part of an upcoming WHO report from the consensus meeting on the assessment of anti-malarial drug exposure in clinical trials). Plasma samples were analysed using high throughput liquid chromatography with tandem mass spectrometry [25]. Briefly, artemether and dihydroartemisinin were extracted using solid phase extraction (Oasis HLB μ-elution plate, Waters, Milford, USA) and quantified on an API 5000™ triple quadruple mass spectrometer (Applied Biosystems/MDS SCIEX, Foster City, USA), with a TurboV ionisation source (TIS) interface operated in the positive ion mode. Stable isotope labelled artemether and dihydroartemisinin were used as internal standards. Selected reaction monitoring (SRM) was used during quantification; transitions m/z 316-163 (artemether), m/z 320-163 (stable labelled artemether), m/z 302-163 (dihydroartemisinin), and m/z 307-166 (stable labelled dihydroartemisinin). The liquid chromatographic (LC) system was an Agilent 1200 system (Agilent technologies, Santa Clara, USA). Data acquisition and quantification were performed using Analyst 1.4 (Applied Biosystems/MDS SCIEX, Foster City, USA). The lower limit of quantification (LLOQ) for both artemether and dihydroartemisinin was set to 1.43 ng/mL with a limit of detection of 0.5 ng/mL. Triplicates of quality control samples at 3.46 ng/mL, 36 ng/mL, and 375 ng/mL for artemether and dihydroartemisinin were analysed in each batch during routine drug analysis.

### Pharmacokinetic analysis

Pharmacokinetic characterization of artemether and dihydroartemisinin was performed using non-compartmental analysis (WinNonlin 5.0, Pharsight Corporation, Ca, USA). Artemether was assumed to be fully transformed into dihydroartemisinin *in vivo *and the relative difference in molecular weights between dihydroartemisinin and artemether were used to compute the putative dose of administered dihydroartemisinin (i.e. 95.5% of artemether dose) [13]. Pharmacokinetic analysis was performed using the linear-up/log-down trapezoidal method for each individual concentration-time profile. Total drug exposure (AUC_0-∞_) was calculated for each subject with extrapolation to time infinity by C_PRED_/λ_Z_, where C_PRED _is the predicted concentration at the time for the last observed concentration and λ_Z _is the elimination rate constant during the terminal phase. The terminal elimination half-life (t_1/2_) was determined by log-linear regression of 4 to 7 observed concentrations. Maximal concentration (C_MAX_) and time to reach maximal concentration (T_MAX_) were taken directly from the observed data. Oral clearance (CL/F) and apparent oral volume of distribution (V_Z_/F) are also reported.

## Results

The oral formulation of artemether was well-tolerated and no clinical adverse effects were observed in any of the study subjects. Artemether and dihydroartemisinin were quantified in a total of 216 plasma samples in twelve healthy Pakistani subjects after a single oral dose of artemether-lumefantrine (80/480 mg artemether/lumefantrine). Of these, 44 artemether concentrations and 52 dihydroartemisinin concentrations (not including pre-dose) were determined to be below the LLOQ. Total precision (%CV) of quality control samples during analysis were determined to 4.7%, 1.4% and 1.5% for artemether and to 3.0%, 1.6% and 0.7% for dihydroartemisinin in low, mid and high (i.e. 3.46 ng/mL, 36 ng/mL and 375 ng/mL) quality control samples.

**Figure 1:**
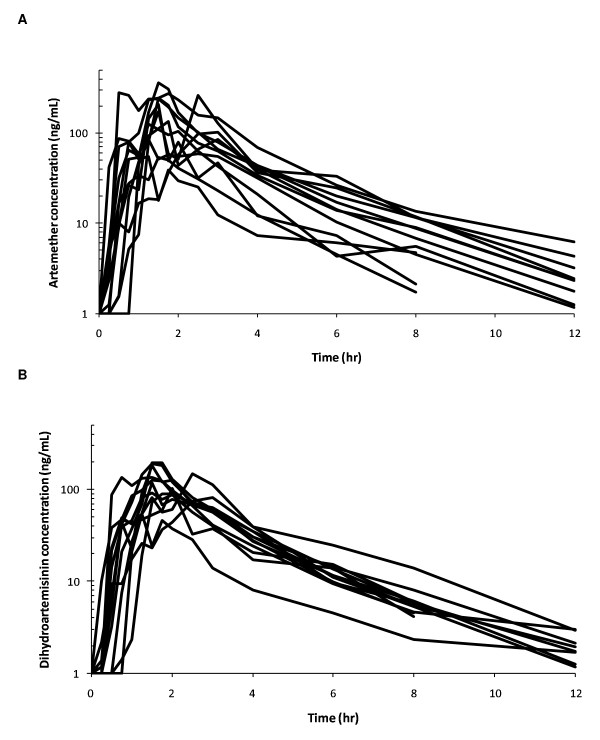
**Pharmacokinetic profiles**. Individual drug plasma concentration-time profiles for artemether (A) and dihydroartemisinin (B) in healthy Pakistani volunteers after a single oral dose of 80 mg artemether and 480 mg lumefantrine. Drug concentrations are plotted on a logarithmic scale with the base 10.

Individual plasma concentration-time profiles were evaluated in all twelve subjects for artemisinin and dihydroartemisinin using non-compartmental analysis (Table 1). Pharmacokinetics was well described but showed large inter-individual variability. One observed concentration was considered an outlier in one subject and removed before analysis (i.e. >10-fold unexplained increase and decrease around data point six hr after dose). Individual artemether and dihydroartemisinin plasma concentration-time curves are shown in Figure 1.

**Table 1:** Non-compartmental analysis of artemether and dihydroartemisinin pharmacokinetics in twelve healthy Pakistani subjects

	Artemether		Dihydroartemisinin	
	**Mean ± SD**	**Median (range)**	**Mean ± SD**	**Median (range)**

Body-weight (kg)	69.9 ± 10	69.0 (51.0-83.0)	69.9 ± 10	69.0 (51.0-83.0)

Dose (mg/kg)	1.17 ± 0.18	1.16 (0.960-1.57)	1.11 ± 0.17	1.10 (0.92-1.49)

T_LAG _(hr)	0.130 ± 0.23	0 (0-0.75)	0.250 ± 0.24	0.250 (0-0.75)

C_MAX _(ng/mL)	184 ± 100	173 (54.2-363)	126 ± 46	122 (53.7-195)

C_MAX_/dose (kg × ng/mL × mg)	160 ± 86	148 (34.6-299)	113 ± 38	107 (57.1-169)

T_MAX _(hr)	1.56 ± 0.68	1.50 (0.500-3.00)	1.69 ± 0.59	1.50 (0.750-3.00)

CL/F (L/hr)	257 ± 140	222 (119-609)	269 ± 57	246 (209-383)

CL/F (L/hr × kg)	3.89 ± 2.8	3.11 (1.57-11.9)	3.87 ± 0.74	3.70 (3.05-5.27)

V_Z_/F (L)	666 ± 220	651 (342-1150)	702 ± 220	643 (456-1190)

V_Z_/F (L/kg)	9.85 ± 4.5	8.68 (5.02-22.5)	10.1 ± 2.6	9.63 (6.61-14.6)

T_1/2 _(hr)	2.00 ± 0.71	1.88 (1.24-4.00)	1.80 ± 0.31	1.78 (1.34-2.20)

AUC_0-LAST _(hr × ng/mL)	379 ± 170	362 (128-664)	287 ± 60	303 (196-361)

AUC_0-∞ _(hr × ng/mL)	385 ± 170	370 (131-671)	294 ± 58	311 (199-365)

AUC_0-∞_/dose (kg × hr × ng/mL × mg)	339 ± 160	322 (83.8-638)	267 ± 48	270 (190-327)

Extrapolated AUC_0-∞ _(%)	1.75 ± 0.99	1.43 (0.90-4.26)	2.59 ± 2.2	1.71 (0.73-8.76)

T_LAG _= absorption lag time, C_MAX _= maximum observed plasma drug concentration, T_MAX _= observed time to reach C_MAX_, CL/F = oral clearance, V_Z_/F = apparent oral volume of distribution based on the terminal phase, T_1/2 _= terminal elimination half-life, AUC_0-LAST _= area under the plasma concentration time curve after the first dose to the last sampling time, AUC_0-∞ _= area under the plasma concentration time curve after the first dose to infinity, and Extrapolated AUC_0-∞ _= percentage of AUC_0-∞ _due to extrapolation from last measured concentration to infinity.

Artemether (80 mg per adult dose) was absorbed rapidly from the gastrointestinal tract with a lag-time of up to 45 minutes. Considerable fluctuations in plasma artemether and dihydroartemisinin concentrations were observed within and between patients resulting in large inter-individual variability in pharmacokinetic parameters. C_MAX _(mean ± SD) for artemether and dihydroartemisinin were 184 ± 100 ng/mL and 126 ± 46 ng/mL, respectively. C_MAX _was reached after 1.56 ± 0.68 hr for artemether and 1.69 ± 0.59 hr for dihydroartemisinin after drug administration. AUC_0-∞_, CL/F, V_Z_/F and t_1/2 _for artemether were 385 ± 170 hr × ng/mL, 257 ± 140 L/hr, 666 ± 220 L, and 2.00 ± 0.71 hr, respectively, while computed parameters for dihydroartemisinin were 294 ± 58 hr × ng/mL, 269 ± 57 L/hr, 702 ± 220 L, and 1.80 ± 0.31 hr, respectively. The mean percentage of total AUC_0-∞ _extrapolated to infinity was below 3% for both artemether and dihydroartemisinin.

## Discussion

The concentration-time profiles of artemether and dihydroartemisinin were evaluated in all of the 12 healthy male Pakistani volunteers and showed similar pharmacokinetics as reported in other populations with different ethnicity (Table 2) [19-22]. Artemether shows a large variability within and between different populations. Maximal drug concentration and total drug exposure of artemether and dihydroartemisinin are generally comparable between the different healthy populations. However, Na-Bangchang *et al *[20] report a slightly longer median artemether terminal elimination half-life but a more than five-fold longer terminal elimination half-life for dihydroartemisinin in both healthy Thai volunteers and Thai patients than reported in other populations. This results in an approximately 10-fold higher total dose-normalized exposure of dihydroartemisinin [20]. To the best of our knowledge, this long half-life has not been reproduced elsewhere. This could possibly be related to drug assay differences. Contradictory to that report, Lefevre *et al *[26] report similar maximum concentration and total drug exposure in 25 Thai patients with uncomplicated *falciparum *malaria as seen in the healthy Pakistani volunteers in this study. Total drug exposure of artemether and dihydroartemisinin was somewhat higher in Chinese patients (n = 22) compared to the healthy volunteers in this study [21]. Artemether is rapidly absorbed from the gastrointestinal tract in all populations and reached maximum concentrations within two hr. An almost simultaneous detection of the artemether and dihydroartemisinin in plasma indicates a rapid *in vivo *conversion of artemether into its active metabolite dihydroartemisinin.

**Table 2:** Pharmacokinetic parameter estimates for artemether and dihydroartemisinin in different ethnic populations after artemether administration

	**Pakistani male healthy volunteers (n = 12)**. Oral single dose of 80 mg artemether ^a,b^ [present study]	Caucasian male and female healthy volunteers **(n = 16)**. **Oral single dose of 80 mg artemether ^a,b ^**[19]	**Thai male healthy volunteers (n = 6)**. **Oral single dose of 200 mg artemether ^c ^**[20]	Malaysian male healthy volunteers **(n = 6)**. **Oral single dose of 200 mg artemether ^c ^**[22]	Chinese male and female patients with malaria **(n = 11)**. **Oral multiple doses of 80 mg artemether ^c,d ^**[21]	Chinese male and female patients with malaria **(n = 11)**. **Oral multiple doses of 80 mg artemether ^a,c,d ^**[21]
***Artemether***						

C_MAX _(ng/mL)	184 ± 100	104 ± 40	118 (112-127)	310 ± 150	157 ± 93 ^e^	133 ± 49 ^e^

T_MAX _(hr)	1.56 ± 0.68	2.00 (1.00-4.00)	3.00 (1.00-10.0)	1.88 ± 0.21	1.73 ± 0.46 ^e^	2.13 ± 0.49 ^e^

CL/F (L/hr)	257 ± 140	-	-	365 ± 190	-	-

V/F (L)	666 ± 220	-	-	988 ± 520	543 ± 950 ^e^	189 ± 130 ^e^

T_1/2 _(hr)	2.00 ± 0.71	1.90 ± 0.80	3.10 (1.00-9.60)	2.00 ± 0.59	1.16 ± 0.44	0.860 ± 0.26

T_LAG _(hr)	0.130 ± 0.23	-	-	0.330 ± 0.13	0.480 ± 0.24 ^e^	0.830 ± 0.37 ^e^

AUC_0-∞_/dose (hr × ng/mL/mg)	4.82 ± 2.2	4.00 ± 1.7	5.50 (1.65-22.2)	3.36 ± 1.4	5.73 ± 3.1	6.36 ± 2.5

AUC_0-∞ _(hr × ng/mL)	385 ± 170	320 ± 140	1100 (330-4440)	671 ± 270	1830 ± 1000	2040 ± 800

						

***Dihydro-artemisinin***						

C_MAX _(ng/mL)	126 ± 46	104 ± 45	379 (162-702)	273 ± 64	161 ± 63 ^e^	105 ± 42 ^e^

T_MAX _(hr)	1.69 ± 0.59	2.50 (1.00-4.00)	6.00 (2.00-12.0)	1.92 ± 0.13	1.86 ± 0.45 ^e^	2.44 ± 0.44 ^e^

CL/F (L/hr)	269 ± 57	-	-	-	-	-

V/F (L)	702 ± 220	-	-	-	275 ± 230 ^e^	246 ± 99 ^e^

T_1/2 _(hr)	1.80 ± 0.31	2.10 ± 0.90	10.6 (4.70-19.2)	-	0.950 ± 0.42	1.06 ± 0.25

T_LAG _(hr)	0.250 ± 0.24	-	-	0.290 ± 0.10	0.350 ± 0.28 ^e^	0.820 ± 0.54 ^e^

AUC_0-∞_/dose (hr × ng/mL/mg)	3.86 ± 0.76	4.33 ± 1.5	34.5 (4.35-202)	3.94 ± 1.2	9.14 ± 2.0	9.80 ± 4.5

AUC_0-∞ _(hr × ng/mL)	294 ± 58	331 ± 110	6590 (830-38700)	753 ± 230	2930 ± 640	3140 ± 1400

^a ^Fixed oral combination of 80 mg artemether and 120 mg lumefantrine

^b ^administered together with a high-fat meal

^c ^administered on an empty stomach

^d ^administered at 0, 8, 24 and 48 hours

^e ^pharmacokinetic parameter estimates based on the first dose

Values are reported as mean ± SD or as median (range).

Dose normalized exposures were calculated using the published mean or median value for total exposure divided with the mean or median dose.

C_MAX _= maximum observed plasma drug concentration, T_MAX _= observed time to reach C_MAX_, CL/F = oral clearance, V/F = apparent oral volume of distribution, T_1/2 _= terminal elimination half-life, T_LAG _= absorption lag time, and AUC_0-∞ _= area under the plasma concentration time curve after the first dose to infinity.

All enzymes involved in the metabolism of drugs are regulated by genes. Because of evolutionary and environmental factors, there is a remarkable degree of genetic variability built into a population. Thus, the genetic factor represents an important cause of inter-individual variation in drug metabolism. Artemether is metabolized to its active metabolite dihydroartemisinin by the enzymes CYP1A2, CYP2B6 and CYP3A4 in humans [13,14]. CYP3A4 is also expressed in the human small intestine where it may contribute to pre-systemic metabolic conversation of artemether into dihydroartemisinin. The high extraction ratio over the liver of artemether should contribute to a substantial first pass metabolism. A previous study has shown that the plasma concentration of artemether doubles when co-administered with a potent CYP3A4 inhibitor (i.e. ketaconazole). This demonstrates the importance of the CYP3A4 present in the mucosa cells of the gut wall and the liver [19]. A wide variation in total CYP3A4 content exists among individuals which have been attributed to both environmental and genetic factors. Therefore, drugs cleared by this isoform are expected to have highly variable pharmacokinetics [18]. However, the very large influence on pharmacokinetics of food intake considerably outweighs all other sources of variability for this drug. In a previous study, a two-fold increase in relative bioavailability of artemether has been reported when administered with a fatty meal [23]. Food intake should attenuate the large variability in oral bioavailability of artemether and reduce part of the fluctuations seen in the plasma concentration profiles. Artemether-lumefantrine was administered with a glass of milk on an empty stomach followed by a high fat breakfast one hr later in the presented study to maximize the relative bioavailability and prevent erratic absorption profiles in the healthy volunteers. Even so, there was considerable inter-individual variability.

## Conclusions

In conclusion, the pharmacokinetics of artemether and dihydroartemisinin was evaluated in 12 healthy male Pakistani volunteers. Pharmacokinetics were described in this population and showed similar values as reported in other populations.

## Competing interests

The authors declare that they have no competing interests.

## Authors' contributions

SA and MHN planned and conducted the clinical study. JT and NL performed the drug analysis and pharmacokinetic evaluation. All authors participated in writing the paper and have approved the final manuscript.
